# Endothelial Activation and Stress Index—A Novel and Simple Prognostic Tool in Coronary Artery Bypass Grafting

**DOI:** 10.3390/jcm14082857

**Published:** 2025-04-21

**Authors:** Philipp Krombholz-Reindl, Andreas Vötsch, Klaus Linni, Rainald Seitelberger, Roman Gottardi, Michael Lichtenauer, Matthias Hammerer, Elke Boxhammer, Andreas Winkler

**Affiliations:** 1 Department of Cardiovascular and Endovascular Surgery, Paracelsus Medical University Salzburg, 5020 Salzburg, Austria; andreas.voetsch@icloud.com (A.V.); k.linni@salk.at (K.L.); r.seitelberger@salk.at (R.S.); a.winkler@salk.at (A.W.); 2 Department of Cardiovascular Surgery, University Heart Center Freiburg-Bad Krozingen, 79189 Freiburg, Germany; roman.gottardi@gmail.com; 3 Faculty of Medicine, Albert Ludwigs University Freiburg, 79085 Freiburg, Germany; 4 Department of Cardiology, Paracelsus Medical University Salzburg, 5020 Salzburg, Austria; m.lichtenauer@salk.at (M.L.); m.hammerer@salk.at (M.H.);

**Keywords:** endothelial activation and stress index (EASIX), coronary artery bypass grafting, risk assessment

## Abstract

**Objectives:** Risk stratification in coronary artery bypass grafting (CABG) remains challenging despite existing models. The Endothelial Activation and Stress Index (EASIX), originally developed for hematological conditions, has shown promise in various medical fields as a predictor of adverse outcomes. EASIX, calculated from lactate dehydrogenase, creatinine, and platelet count, reflects endothelial dysfunction and systemic inflammation. This study investigates EASIX’s potential in predicting mortality and morbidity in patients undergoing CABG. **Methods:** A total of 475 patients undergoing isolated CABG between January 2017 and June 2020 were retrospectively analyzed. EASIX scores were calculated from pre-operative blood samples. Patients were stratified based on an EASIX cut-off value of 1.16. **Results:** Patients with EASIX ≥ 1.16 were older and had more comorbidities. They experienced higher 30-day mortality (5.0% vs. 0.8%, *p* = 0.004), increased wound infections (6.7% vs. 2.5%, *p* = 0.035), and more frequent prolonged ventilation (9.2% vs. 4.2%, *p* = 0.040). The long-term survival analysis showed significant differences at 3 years (*p* = 0.030) and 5 years (*p* < 0.001). EASIX demonstrated moderate discriminatory power for long-term survival (AUROC 0.669, 95% CI: 0.598–0.740, *p* < 0.001). Importantly, the multivariable analysis revealed EASIX as an independent risk factor for long-term mortality, even after adjusting for traditional risk factors and comorbidities (HR: 2.65, 95% CI: 1.59–4.42, *p* < 0.001). **Conclusions:** EASIX ≥ 1.16 was associated with postoperative morbidity and poorer long-term survival in patients undergoing CABG. This easily calculable score could enhance risk stratification and guide personalized postoperative management.

## 1. Introduction

The Endothelial Activation and Stress Index (EASIX) has emerged as a promising prognostic tool across various medical disciplines. This composite index, derived from serum lactate dehydrogenase (LDH), platelet count, and creatinine levels, serves as a surrogate marker for systemic inflammation and renal stress.

Originally developed as a prognostic marker in hematological malignancies, EASIX was first utilized in patients undergoing allogeneic hematopoietic stem-cell transplantation (alloSCT), where an EASIX score ≥ 3 was associated with a more than twofold increase in transplant-related mortality [1]. Beyond alloSCT, EASIX has demonstrated prognostic value in diffuse large B-cell lymphoma [2] and multiple myeloma [3].

Beyond hematology, EASIX has gained relevance in infectious diseases, particularly during the COVID-19 pandemic, where it was proposed as a simple and universal prognostic index for survival in infected patients [4,5]. More recently, its applicability has extended to traumatic brain injury, where it has been identified as an effective predictor of mortality [6].

Preoperative risk assessment plays an important role in optimizing outcomes for patients undergoing coronary artery bypass grafting (CABG). Given the complexity and inherent risks associated with cardiac surgery, a comprehensive evaluation that integrates clinical scoring systems, biomarkers, and frailty assessments is essential.

Traditional risk models, such as the Society of Thoracic Surgeons (STS) Score and EuroSCORE II, have long been established as the cornerstone of preoperative evaluation in cardiac surgery [7,8].

In addition to traditional risk scores, circulating biomarkers may offer valuable complementary insights. Biomarkers such as high-sensitivity troponin, natriuretic peptides, and C-reactive protein have been associated with myocardial injury, hemodynamic stress, and systemic inflammation, respectively [9], and have shown predictive value for prognosis in cardiovascular disease [10,11,12]. Preoperative frailty assessment, with systemic inflammation regarded as a key pathogenetic mechanism, has become increasingly important in risk stratification prior to CABG [13,14].

A study published in 2024 highlighted its potential as a predictor of mortality in coronary artery disease (CAD) patients. Elevated EASIX predicted independently mortality in CAD [15].

The analyzed cohort did not include patients undergoing CABG, a group typically characterized by more extensive macroangiopathy. The prognostic value of EASIX demonstrated in this study pertains specifically to patients with less advanced disease managed medically or with percutaneous coronary intervention (PCI) and may not be generalizable to surgically treated populations.

The EASIX-Score is derived from routine laboratory parameters; it offers a cost-effective, objective, and easily measurable approach to evaluate inflammation, endothelial stress, and renal function, and therefore, it might be an interesting tool in preoperative risk stratification.

## 2. Material and Methods

From January 2017 to June 2020, 475 consecutive patients scheduled for elective or urgent isolated CABG at the Department of Cardiac, Vascular, and Endovascular Surgery at Paracelsus Medical University in Salzburg, Austria, were enrolled in the study. Patients with acute coronary syndrome with ST elevation myocardial infarction (STEMI), unstable angina as defined in the (ESC) Guidelines for the management of acute coronary syndromes [16], and patients requiring concomitant procedures were excluded in advance to ensure a homogenous cohort. Patient demographics are summarized in Table 1.

The local ethics committee of the state of Salzburg granted approval for the study under protocol number 1168/2020 on 25 November 2020. Data management adhered to the ethical principles set forth in the Declaration of Helsinki and the (ICH-GCP) guidelines (International Council for Harmonisation of Technical Requirements for Pharmaceuticals for Human Use—Good Clinical Practice). The requirement for informed consent was waived.

Patient information was obtained from the ORBIS electronic medical records system (Version 08043301.04110DACHL, Dedalus Healthcare GmbH, Bonn, Germany). Long-term mortality was assessed with follow-up calls.

### 2.1. Patient Sample Collection and EASIX Score

Blood samples from patients were collected 1–3 days before CABG surgery. All samples were obtained following standard venipuncture procedures and processed immediately to ensure the stability of key biomarkers. Laboratory parameters relevant to the EASIX score were measured using standardized clinical chemistry assays in an accredited laboratory.

The Endothelial Activation and Stress Index (EASIX) was calculated using routine laboratory parameters, specifically lactate dehydrogenase (LDH), creatinine, and platelet count (EASIX = LDH (U/L) × Creatinine (mg/dL)/Platelet Count (10^9^/L)).

### 2.2. Surgical Procedure

The indication for CABG was ischemic heart disease, determined by a specialized multidisciplinary heart team consisting of a cardiac surgeon, cardiologist, and cardiac anesthesiologist in accordance with current guidelines [17]. The procedures were performed either by senior surgeons or by residents under supervision. Most patients underwent median sternotomy under general anesthesia. Graft selection and perioperative medication management followed established guideline recommendations [18].

## 3. Statistics

All statistical analyses and data visualizations were performed using the SPSS software (Version 25.0, SPSS Inc., Armonk, NY, USA). The Kolmogorov–Smirnov–Lilliefors test was utilized to assess the distribution of variables. Continuous variables exhibiting a normal distribution are presented as mean ± standard deviation (SD) and were compared using an unpaired Student’s t-test. For variables that do not follow a normal distribution, data are expressed as the median with an interquartile range (IQR) and were analyzed via the Mann–Whitney U test. Categorical variables are reported as frequencies or percentages and were evaluated using the chi-squared test. Patients were divided into quartiles based on their EASIX values, with individuals in the highest quartile identified as particularly vulnerable to increased overall mortality and cardiovascular events.

To assess the predictive value of EASIX for long-term outcomes in CABG surgery, an area under the receiver operating characteristic (AUROC) curve was generated. The area under the curve (AUC), sensitivity, specificity, and Youden Index (YI) was determined. Univariate Cox regression analysis was employed to investigate whether various clinical characteristics (e.g., sex, age, diabetes) were associated with an elevated risk of mortality in relation to AS severity.

A Kaplan–Meier survival analysis was conducted to examine differences in short- and long-term mortality after CABG based on varying EASIX cut-off values, with log-rank tests and corresponding risk numbers included. Additionally, a univariate Cox regression analysis was performed to identify clinical factors associated with long-term mortality across the patient cohort. To enhance comparability, metric data underwent z-transformation. Variables with a *p*-value ≤ 0.100 in univariate analysis were incorporated into a subsequent multivariable Cox regression model to determine independent mortality predictors, utilizing backward elimination for model refinement.

A *p*-value of < 0.050 was regarded as statistically significant in all analyses.

A comprehensive post hoc power analysis using G*Power 3.1 [19] confirmed that the study achieved a statistical power exceeding 95%, considering an allocation ratio of 3:1. This high-power level underscores the study’s ability to detect meaningful differences or significant effects between groups, thereby minimizing the risk of a Type II error (failing to identify an existing effect). The analysis was conducted with a conventional alpha (α) level of 0.05, the standard threshold for statistical significance, assuming a medium effect size (d = 0.5).

## 4. Results

### 4.1. Determination of Cut-Off Value EASIX ≥ 1.16

Due to lack of evidence for a reliable cut-off value in our study population, we explored a potential cut-off value with two methods: Patients were stratified into quartiles based on their EASIX scores. The higher quartile were patients with an EASIX score of 1.16 and above.

**Figure 1:**
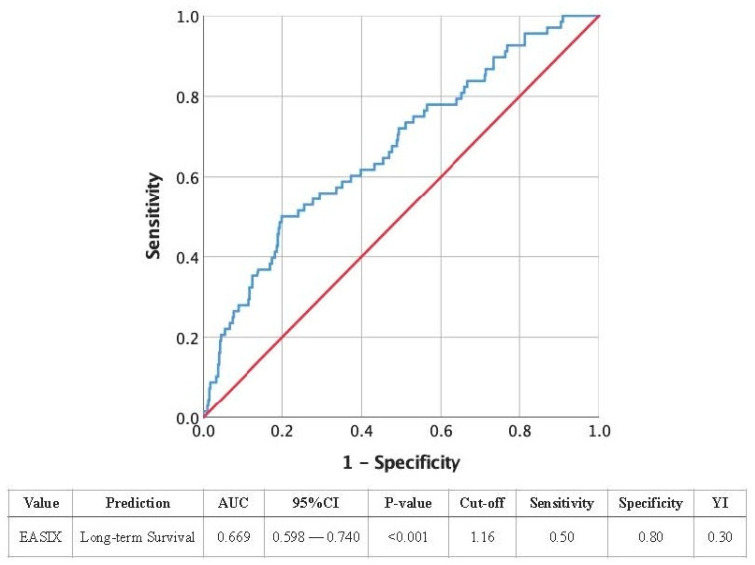
The AUROC curve for EASIX as a predictor of long-term survival following CABG.

Furthermore, an AUROC curve analysis for EASIX as a predictor of long-term survival following CABG was conducted (Figure 1). The AUC was 0.669 (95% CI: 0.598–0.740, *p* < 0.001), indicating a moderate discriminative ability. The optimal cut-off value for the EASIX score was determined to be 1.16—identical to the value of the calculated higher quartile of the study cohort—with a sensitivity of 0.50 and a specificity of 0.80. The Youden Index (YI) was calculated at 0.30, indicating a moderate ability of the EASIX score to discriminate between patients regarding to survival outcomes.

### 4.2. Baseline Characteristics

Male patients represented the majority of the cohort (79.8%), with no significant difference in sex distribution between the groups (*p* = 0.167). Patients with an EASIX ≥ 1.16 were older (74 vs. 68 years *p* < 0.001), were more likely to have peripheral artery disease (PAOD), 20.8% vs. 9.6%, *p* = 0.001), atrial fibrillation (AF, 15.0% vs. 6.5%, *p* = 0.004), and prior myocardial infarction (MI, 42.5% vs. 31.3%, *p* = 0.025).

As expected, renal function and related parameters included in the EASIX score were significantly different.

The EuroSCORE II was significantly higher in the higher EASIX group (2.6 ± 3.1 vs. 1.5 ± 1.4, *p* < 0.001).

Further baseline characteristics are shown in Table 1.

### 4.3. Intraoperative Data

There was no significant difference regarding the use of the heart–lung machine (*p* = 0.063) between the groups. Aortic cross-clamp time was shorter (45.0 ± 27.5 min vs. 50.0 ± 28.0 min, *p* = 0.028), arterial bypass grafts were fewer (median: 1.0 vs. 2.0, *p* = 0.001), and venous bypass grafts were more frequent (median: 2.0 vs. 1.0, *p* = 0.030) in the EASIX ≥ 1.16 group. Table 2 shows the intraoperative data.

### 4.4. Postoperative Outcomes

Patients with EASIX levels ≥ 1.16 demonstrated a significantly higher 30-day mortality compared to those with EASIX < 1.16 (5.0% vs. 0.8%; *p* = 0.004). The incidence of wound infections was significantly higher in the EASIX ≥ 1.16 group (6.7% vs. 2.5% *p* = 0.035) as well as prolonged ventilation (9.2% vs. 4.2% *p* = 0.040) and ICU stay (standard criteria for discharging were applied and followed by the staff, *p*= 0.002). There was no difference regarding the incidence of myocardial infarction defined as type 5 myocardial infarction (myocardial infarction associated with coronary artery bypass grafting according to the 4th universal definition of myocardial infarction, 1.1% vs. 3.3% *p* = 0.104) and major neurological events (modified Rankin score ≥ 2 at 90 days postoperatively 1.7% vs. 1.7% *p* = 0.986). Other postoperative outcome parameters are shown in Table 3.

### 4.5. Mid- and Long-Term Outcomes

**Figure 2:**
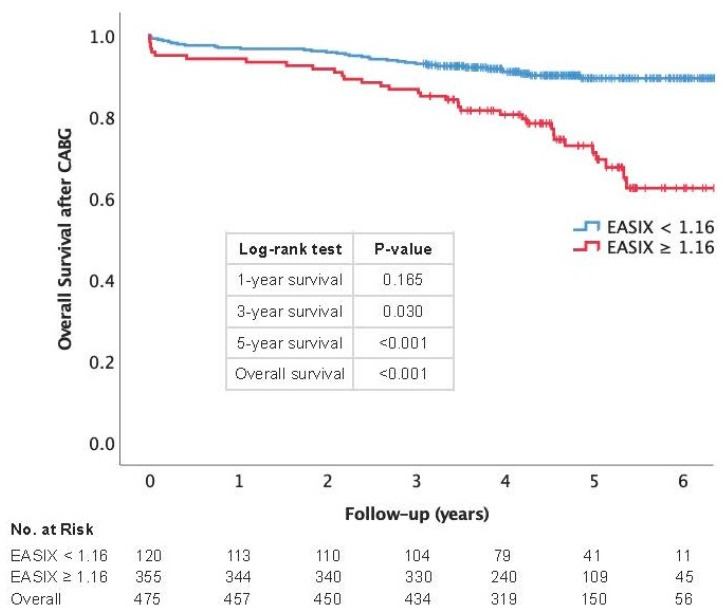
A Kaplan–Meier survival curve demonstrating a significant lower overall survival in patients with EASIX ≥ 1.16.

Figure 2 shows a Kaplan–Meier survival curve demonstrating a significant difference in overall survival between patients with EASIX scores below and above the cut-off value of 1.16. While 1-year survival rates were not significantly different (*p* = 0.165), survival differences became evident at 3 years (*p* = 0.030) and were highly significant at 5 years (*p* < 0.001). Overall survival across the entire follow-up period was significantly worse for patients with an EASIX score ≥ 1.16 (log-rank *p* < 0.001).

**Figure 3:**
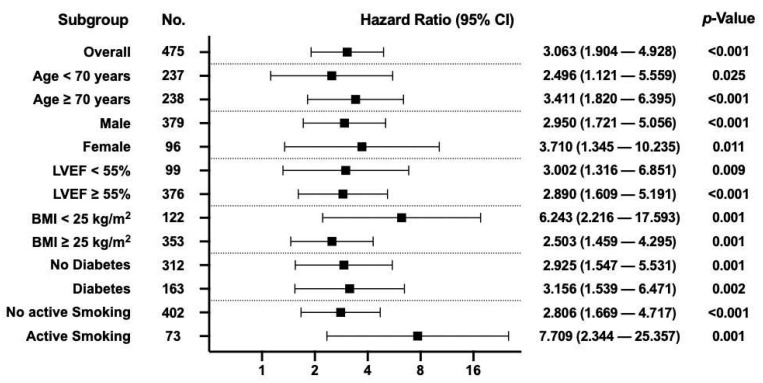
A Cox proportional hazards regression analysis, evaluating HR for overall mortality across several subgroups in patients with EASIX ≥ 1.16.

Figure 3 presents a Cox proportional hazards regression analysis, evaluating HR for overall mortality across several subgroups in patients with EASIX ≥ 1.16. Independent of the selected patient characteristics, a consistently significant increase in HR values was observed for an EASIX score ≥ 1.16. Notably, even patients younger than 70 years had a 2.5-fold higher risk of long-term mortality after CABG when their EASIX score exceeded 1.16 (*p* = 0.025).

### 4.6. EASIX as Independent Risk Factor for Long-Term Mortality in CABG

Another Cox proportional hazard regression analysis, presented in Table 4, identified several factors associated with general long-term mortality in patients after CABG. EASIX emerged as a significant independent risk factor for long-term mortality, with a HR of 3.063 (95% CI: 1.904–4.928, *p* < 0.001) in the univariate analysis, which remained robust in the multivariable model (HR: 2.652, 95% CI: 1.593–4.415, *p* < 0.001). Age was significant in the univariate analysis (HR: 1.420, 95% CI: 1.086–1.856, *p* = 0.010), but this association did not persist after adjusting for other variables (HR: 1.109, 95% CI: 0.795–1.549, *p* = 0.542), suggesting it is confounded by other clinical factors. Other significant predictors in the multivariable model included BMI (HR: 1.336, 95% CI: 1.027–1.738, *p* = 0.031), hemoglobin (HR: 0.725, 95% CI: 0.586–0.897, *p* = 0.003), and LVEF (HR: 1.379, 95% CI: 1.077–1.767, *p* = 0.001), while variables such as EuroSCORE II, arterial hypertension, and prior comorbidities lost significance after adjustment.

## 5. Discussion

This study demonstrates that EASIX might be a valuable prognostic tool for predicting long-term mortality. In our cohort, an EASIX score ≥ 1.16 was associated with significantly higher 30-day mortality, increased incidence of wound infections, prolonged ventilation and ICU stay, as well as worse long-term survival outcomes increasingly apparent at 3 and 5 years post-CABG.

Initially designed for hematological disorders [1,2,3], the EASIX score has demonstrated exceptional adaptability across multiple medical fields. Our study extends its applicability to coronary artery disease undergoing CABG procedure, where it demonstrates a predictive value for both short-term complications and long-term mortality. The AUROC of 0.669 for long-term survival prediction indicates moderate discriminatory power, suggesting that EASIX could be a useful addition to existing risk stratification tools in CABG patients.

Patients with EASIX ≥ 1.16 were typically older and had a higher prevalence of comorbidities such as peripheral artery disease, atrial fibrillation, and prior myocardial infarction. This aligns with the understanding that endothelial dysfunction and systemic inflammation, which EASIX aims to quantify, are more prevalent in older individuals and those with multiple cardiovascular risk factors [20,21]. The higher EuroSCORE II values in this group further confirm their increased surgical risk profile [8,22].

Intraoperatively, patients with elevated EASIX scores received fewer bilateral internal mammary artery (BIMA) grafts, which may reflect surgeons’ preference for less extensive procedures in higher-risk patients. The shorter cross-clamping times and fewer arterial bypasses in this group could also be attributed to a more conservative surgical approach in patients perceived to be at higher risk.

The predictive capability of EASIX for short-term survival was recently demonstrated by Sang et al. [23] in a cohort of coronary heart disease patients with over 1000 myocardial infarction patients. Notably, the calculated cut-off values were considerably higher than those in the present study, likely due to the fundamentally different patient populations analyzed (ST elevation myocardial infarction and patients with ongoing ischemia were excluded in our study).

The consistent elevation in hazard ratios across various subgroups for patients with EASIX ≥ 1.16 suggests that the prognostic value of EASIX is independent of these traditional risk factors. These findings align with the study by Finke et al. [15]. Elevated EASIX score was an independent prognostic marker for overall survival before and after coronary catheterization. Importantly, these effects remained significant after adjusting for similar confounding factors such as age, diabetes, and left ventricular function, emphasizing EASIX’s ability to reflect systemic endothelial stress beyond conventional risk factors, as it was shown in our study.

Due to its simplicity in calculation, relying on routinely collected laboratory parameters, the EASIX score might be an attractive option for clinical implementation. Future research should focus on validating these findings in larger, multi-center prospective studies and exploring how EASIX can be integrated with existing risk stratification tools to enhance perioperative decision-making and long-term patient management in cardiac surgery.

## 6. Limitations of the Study

This study has several limitations that should be acknowledged. First, its retrospective, single-center design introduces inherent risks of selection bias and limits the generalizability of the findings to other institutions or patient populations. Second, emergency procedures and patients undergoing concomitant cardiac surgeries were excluded, which restricts the applicability of the results to elective, isolated CABG cases. Third, while the EASIX score demonstrated moderate predictive value for short- and long-term outcomes, the study did not assess its integration with established risk models, such as EuroSCORE II or the STS risk score. Fourth, an internal validation was not conducted, which limits our ability to assess the robustness and potential overfitting of the model within our dataset. Moreover, external validation in independent, multi-center cohorts was not performed, which is necessary to confirm the robustness and transferability of these findings. Prospective studies are warranted to address these limitations and to evaluate the potential role of EASIX in enhancing existing risk stratification tools in cardiac surgery.

## Figures and Tables

**Table 1:** Baseline characteristics between the low and high EASIX score group.

	Total	EASIX < 1.16	EASIX ≥ 1.16	*p*-Value
No. (%)				
Total	475 (100.0)	355 (74.7)	120 (25.3)	-
Sex (male)	379 (79.8)	278 (78.3)	101 (84.2)	0.167
Age > 70 years	238 (50.1)	158 (44.5)	80 (66.7)	**<0.001**
BMI ≥ 25.0 kg/m^2^	353 (74.3)	259 (73.0)	94 (78.3)	0.244
Arterial Hypertension	428 (90.1)	318 (89.6)	110 (91.7)	0.508
Diabetes Mellitus	163 (34.3)	117 (33.0)	46 (38.3)	0.284
Dyslipidemia	421 (88.6)	311 (87.6)	110 (91.7)	0.226
Familiar History of CV Disease	123 (25.9)	99 (27.9)	24 (20.0)	0.088
PAOD	59 (12.4)	34 (9.6)	25 (20.8)	**0.001**
COPD				0.782
I°	51 (10.7)	36 (10.1)	15 (12.5)	
II°	16 (3.4)	12 (3.4)	4 (3.3)	
III°	5 (1.1)	3 (0.8)	2 (1.7)	
AF	41 (8.6)	23 (6.5)	18 (15.0)	**0.004**
Active Smoker	73 (15.4)	64 (18.0)	9 (7.5)	**0.006**
Prior Stroke	43 (9.1)	29 (8.2)	14 (11.7)	0.248
Prior MI	162 (34.1)	111 (31.3)	51 (42.5)	**0.025**
eGFR				**<0.001**
≥90 mL/min/BSA	193 (40.3)	175 (49.3)	18 (15.0)	
60–89 mL/min/BSA	233 (49.1)	170 (47.9)	63 (52.5)	
31–59 mL/min/BSA	40 (8.4)	10 (2.8)	30 (25.0)	
≤30 mL/min/BSA	9 (1.9)	0 (0.0)	9 (7.5)	
NYHA				0.684
I	138 (29.1)	108 (30.4)	30 (25.0)	
II	182 (38.3)	135 (38.0)	47 (39.2)	
III	88 (18.5)	63 (17.7)	25 (20.8)	
IV	67 (14.1)	49 (13.8)	18 (15.0)	
LVEF				0.317
≥55%	376 (79.2)	287 (80.8)	89 (74.2)	
41–54%	56 (11.8)	37 (10.4)	19 (15.8)	
31–40%	22 (4.6)	17 (4.8)	5 (4.2)	
≤30%	21 (4.4)	14 (3.9)	7 (5.8)	
**Median ± IQR**				
Age (years)	70.0 ± 14.0	68.0 ± 14.0	74.0 ± 11.0	**<0.001**
Height (cm)	172.0 ± 11.0	172.0 ± 11.0	172.0 ± 11.0	0.928
Weight (kg)	82.0 ± 18.0	82.0 ± 19.0	80.5 ± 19.0	0.777
BMI (kg/m^2^)	27.9 ± 5.5	27.5 ± 5.6	27.3 ± 5.5	0.551
EuroSCORE II	1.6 ± 1.9	1.5 ± 1.4	2.6 ± 3.1	**<0.001**
Creatinine (mg/dl)	1.0 ± 0.3	0.9 ± 0.2	1.3 ± 0.5	**<0.001**
eGFR (mL/min/BSA)	77.4 ± 38.6	83.3 ± 37.8	59.4 ± 33.2	**<0.001**
Platelet Count (10^9^/L)	229.0 ± 77.0	242.0 ± 71.0	186.0 ± 65.8	**<0.001**
LDH (U/L)	204.0 ± 50.0	198.0 ± 42.0	226.5 ± 70.0	**<0.001**
CRP (mg/dL)	0.2 ± 0.5	0.2 ± 0.4	0.4 ± 0.8	**<0.001**
Hemoglobin (g/dL)	14.2 ± 2.1	14.3 ± 1.9	13.7 ± 2.2	**0.001**

Values are presented as mean ± standard deviation and % (*n*) and median ± IQR. *p*-values are two-sided. BMI = Body Mass Index. CV = Cardiovascular. COPD = Chronic Obstructive Pulmonary Disease. LVEF = Left Ventricle Ejection Fraction. NYHA = New York Heart Association. PAOD = Peripheral Obstructive Artery Disease. eGFR = Estimated Glomerular Filtration Rate. LDH = Lactate Dehydrogenase. CRP = C-reactive Protein. Bold values are statistically significant.

**Table 2:** The intraoperative data of the low EASIX group vs. high EASIX group.

	Total	EASIX < 1.16	EASIX ≥ 1.16	*p*-Value
**No. (%)**				
On-pump CABG	435 (91.6)	330 (93.0)	105 (87.5)	0.063
CABG via Left Thoracotomy	4 (0.8)	2 (0.6)	2 (1.7)	0.839
ITA Configuration				**<0.001**
None	41 (8.6)	25 (7.0)	16 (13.3)	
LIMA	246 (51.8)	175 (49.3)	71 (59.2)	
RIMA	35 (7.4)	28 (7.9)	7 (5.8)	
BIMA	153 (32.2)	127 (35.8)	26 (21.7)	
IABP used	7 (1.6)	5 (1.4)	2 (1.7)	0.253
ECMO used	6 (1.3)	5 (1.4)	1 (0.8)	0.626
**Mean ± SD**				
Intraoperative packed red blood cells/patient	0.3 ± 0.9	0.2 ± 0.9	0.4 ± 0.7	0.177
**Median ± IQR**				
Perfusion Time (min)	93.0 ± 49.0	94.0 ± 51.0	88.0 ± 45.8	0.090
Aortic Cross-clamp Time (min)	49.0 ± 27.0	50.0 ± 28.0	45.0 ± 27.5	**0.028**
Arterial Bypass Grafts	2.0 ± 1.0	2.0 ± 1.0	1.0 ± 1.0	**0.001**
Venous Bypass Grafts	1.0 ± 2.0	1.0 ± 2.0	2.0 ± 1.8	**0.030**

Values are presented as mean ± standard deviation, % (*n*), and median ± IQR. *p*-values are two-sided. IQR= Interquartile Range. ITA = Internal Thoracic Artery, LIMA = Left Internal Mammary Artery, BIMA = Bilateral Internal Mammary Artery, RIMA = Right Internal Mammary Artery, ECMO = Extracorporeal Membrane Oxygenation = Extracorporeal Life Support, IABP = Intra-aortic Balloon Pump. Bold values are statistically significant.

**Table 3:** The in-hospital outcome parameters between the low vs. high EASIX group.

	Total	EASIX < 1.16	EASIX ≥ 1.16	*p*-Value
**No. (%)**				
30-day mortality	9 (1.9)	3 (0.8)	6 (5.0)	**0.004**
Major Neurological Events	8 (1.7)	6 (1.7)	2 (1.7)	0.986
Perioperative MI	8 (1.7)	4 (1.1)	4 (3.3)	0.104
AKI requiring Hemodialysis	7 (1.5)	3 (0.8)	4 (3.3)	0.051
Sternal Wound Infection	17 (3.6)	9 (2.5)	8 (6.7)	**0.035**
AF (new onset)	109 (22.9)	78 (22.0)	31 (25.8)	0.384
Reoperation due to Bleeding/Tamponade	8 (1.7)	7 (2.0)	1 (0.8)	0.402
Prolonged Ventilation ≥ 48 h	26 (5.5)	15 (4.2)	11 (9.2)	**0.040**
Reintubation Rate	17 (3.6)	11 (3.1)	6 (5.0)	0.332
Reoperation/Reintervention due to Graft occlusion	7 (1.5)	4 (1.1)	3 (2.5)	0.280
**Median ± IQR**				
Invasive Ventilation Time (h)	5.0 ± 4.0	5.0 ± 3.0	6.0 ± 5.0	**0.013**
Intensive Care Unit Stay (d)	2.0 ± 2.0	2.0 ± 2.0	2.0 ± 3.8	**0.002**

Values are presented as mean ± standard deviation, % (*n*), and median ± IQR. *p*-values are two-sided. MI = Myocardial Infarction. AKI = Acute Kidney Injury. AF = Atrial Fibrillation. IQR = Interquartile Range. Bold values are statistically significant.

**Table 4:** A univariate and multivariable Cox proportional hazard regression analysis.

Cox Regression Analysis	Univariate		Multivariable	
	Hazard Ratio (95% CI)	*p*-Value	Hazard Ratio (95% CI)	*p*-Value
Long-Term Mortality				
EASIX	3.063 (1.904–4.928)	<0.001	2.652 (1.593–4.415)	**<0.001**
Sex (male)	0.892 (0.502–1.582)	0.695		
Age	1.420 (1.086–1.856)	0.010	1.109 (0.795–1.549)	0.542
Weight	0.952 (0.750–1.208)	0.685		
Height	1.201 (0.955–1.510)	0.117		
BMI	1.238 (1.000–1.533)	0.050	1.336 (1.027–1.738)	**0.031**
EuroSCORE II	1.389 (1.226–1.573)	<0.001	1.017 (0.808–1.280)	0.886
Arterial Hypertension	7.494 (1.040–53.985)	0.046	4.345 (0.600–31.455)	0.146
Diabetes mellitus	1.565 (0.970–2.526)	0.067	1.214 (0.716–2.058)	0.472
Dyslipidemia	1.201 (0.549–2.628)	0.646		
Family History of CV Disease	0.789 (0.445–1.400)	0.419		
Active Cancer	2.258 (1.076–4.739)	0.031	2.152 (0.974–4.752)	0.058
PAOD	2.513 (1.434–4.404)	0.001	1.410 (0.724–2.745)	0.312
COPD	1.381 (0.983–1.940)	0.063	0.990 (0.675–1.453)	0.961
Prior Stroke	1.716 (0.850–3.464)	0.132		
Prior MI	1.379 (0.851–2.235)	0.192		
Active Smoker	1.205 (0.631–2.301)	0.572		
Atrial fibrillation	2.038 (1.041–3.990)	0.038	1.495 (0.700–3.193)	0.299
Left Main Lesion	1.251 (0.777–2.014)	0.357		
NYHA	1.570 (0.969–2.546)	0.067	1.189 (0.901–1.571)	0.221
eGFR	0.623 (0.475–0.817)	0.001	0.761 (0.544–1.064)	0.110
CRP	1.164 (0.982–1.379)	0.079	1.165 (0.959–1.416)	0.125
Hemoglobin	0.768 (0.654–0.901)	0.001	0.725 (0.586–0.897)	**0.003**
LVEF	0.475 (0.287–0.785)	0.004	1.379 (1.077–1.767)	**0.011**
Mitral Valve Regurgitation	1.400 (0.970–2.019)	0.072	0.946 (0.618–1.449)	0.800
Tricuspid Valve Regurgitation	1.281 (0.855–1.919)	0.230		

Values are presented as hazard ratio (confidence interval). BMI = Body Mass Index. CV = Cardiovascular. COPD = Chronic Obstructive Pulmonary Disease. MI = Myocardial Infarction. LVEF = Left Ventricle Ejection Fraction. NYHA = New York Heart Association. PAOD = Peripheral Obstructive Artery Disease. eGFR = Estimated Glomerular Filtration Rate. LDH = Lactate Dehydrogenase. CRP = C-reactive Protein. Bold values are statistically significant.

## Data Availability

The data underlying this article will be shared upon reasonable request to the corresponding author.

## Abbreviations

AF	Atrial Fibrillation
alloSCT	Allogeneic Hematopoietic Stem-Cell Transplantation
AUC	Area Under the Curve
CABG	Coronary Artery Bypass Grafting
CAD	Coronary Artery Disease
COPD	Chronic Obstructive Pulmonary Disease
CV	Cardiovascular
EASIX	Endothelial Activation and Stress Index
eGFR	Estimated Glomerular Filtration Rate
ESC	European Society of Cardiology
HR	Hazard Ratio
ICH-GCP	International Council for Harmonisation of Technical Requirements for Pharmaceuticals for Human Use—Good Clinical Practice
LDH	Lactate Dehydrogenase
LVEF	Left Ventricular Ejection Fraction
MI	Myocardial Infarction
NYHA	New York Heart Association
PAOD	Peripheral Arterial Obstructive Disease
PCI	Percutaneous Coronary Intervention
SD	Standard Deviation
STEMI	ST elevation myocardial infarction
YI	Youden Index
